# Rifampin- and Multidrug-Resistant Tuberculosis in Russian Civilians and Prison Inmates: Dominance of the Beijing Strain Family

**DOI:** 10.3201/eid0811.020507

**Published:** 2002-11

**Authors:** Francis Drobniewski, Yanina Balabanova, Michael Ruddy, Laura Weldon, Katya Jeltkova, Timothy Brown, Nadezdna Malomanova, Elvira Elizarova, Alexander Melentyey, Ebgeny Mutovkin, Svetlana Zhakharova, Ivan Fedorin

**Affiliations:** * Guy's King's and St Thomas' Medical School, King's College Hospital, London, United Kingdom; † Central Tuberculosis Research Institute, Moscow, Russian Federation; ‡ Samara Oblast Dispensary, Samara City, Russian Federation; § Samara Prison TB Service, Samara City, Russian Federation

## Abstract

Consecutive patient cultures (140) of Mycobacteriium tuberculosis were collected from five Russian civilian and prison tuberculosis laboratories and analyzed for rifampin (rpoB) and isoniazid resistance (inhA, katG, ahpC); transmission of Beijing family isolates; and the importance of prison and previous therapy in drug resistance. Rifampin, isoniazid, and multidrug resistance occurred in 58.2%, 51.6%, and 44.7% of cultures, respectively; 80% of prison cultures were rifampin resistant. Spoligotyping and variable number tandem repeat (VNTR) fingerprinting divided the isolates into 43 groups. Spoligotyping demonstrated that a high proportion (68.1%) of patients were infected with Beijing family strains and that most (69.1%) were rifampin resistant; the highest proportion (81.6%) occurred in prison. One VNTR subgroup (42435) comprised 68 (72.3%) of the Beijing isolates with a small number of IS6110 types; 50 (73.5%) were rifampin resistant. Rifampin-resistant Beijing isolates are dominant within the patient population, especially among prisoners, and threaten treatment programs.

The true extent of drug-resistant tuberculosis (TB) globally is unknown. Few national surveys are representative and validated by external laboratories (1,2). Accurate drug-susceptibility testing is difficult to do well. The quality of results can be further compromised if bacterial cultures originate from a selected population from which key groups, such as prisoners, have been omitted. Only limited susceptibility-testing data are available in those countries with the highest rates of TB such as India, Pakistan, Indonesia, China, and (3-6) Russia.

The spread of resistant isolates, especially multidrug-resistant strains that are resistant to at least isoniazid and rifampin, compromises both clinical efficacy and public health control measures. Patients with infectious multidrug-resistant cases continue to expectorate smear-positive sputum longer than patients with drug-sensitive cases, increasing the probability of spread of infection. The transmission of highly drug-resistant strains has been documented within health-care institutions and prisons (), and the spread of one highly resistant strain, W, has been documented from New York City to other U.S. states and Puerto Rico (7).

In Russia, TB incidence rates declined from the 1950s to the 1990s; the lowest incidence and mortality rates were recorded in 1991 (34.0/100 000 and 8.1/100 000, respectively). By 1999, these rates had risen to 85.2/100 000 and 20.0/100 000, respectively (8-10). The average age of TB patients has declined, reflecting high levels of recent transmission. Data on drug resistance for the whole of the Russian Federation are scarce; information comparable with international data have been reported by the World Health Organization from only 2 of the 89 oblasts (regions). In Ivanavo and Tomsk Oblasts, the prevalence of multidrug-resistant TB was 9.0% and 6.5%, respectively, in 1998-1999 (1,2).

We initiated a pilot study in Samara, Russia, 1 of 89 oblasts to determine the following: the value of genotypic methods for identifying rifampin resistance and multidrug-resistant TB; the extent of rifampin and multiple-drug resistance within the civilian and prison systems; the extent to which this drug resistance was associated with dominant strains, such as those of the Beijing family, by using molecular DNA fingerprinting techniques; and whether drug resistance was associated with being a prisoner or with previous TB treatment.

DNA fingerprinting that uses techniques such as restriction fragment length polymorphism (RFLP) based on IS6110 is the international standard for documenting transmission at the molecular level (11-14). Rapid techniques that use polymerase chain reaction (PCR) amplification such as spacer-oligonucleotide typing (spoligotyping) (13-15) are usually less discriminating but may provide greater information on evolutionary origins and can be used when cultures are contaminated or nonviable.

Spoligotyping is particularly valuable in defining strains belonging to the Beijing family as well as subspeciating strains of the Mycobacteriium tuberculosis complex and does not require viable organisms. The Beijing family of isolates was first reported at a high rate in the Beijing area in 1995 (16) and less frequently in other parts of Asia such as Vietnam (17). These isolates may be associated with a higher occurrence of drug resistance; for instance, the highly drug-resistant W strain, first identified in New York, is a member of the Beijing family (7,18). They These isolates have previously been frequently seen in convenience samples taken in the Russian Federation (18,19). In our study, isolates were collected from both the prison and civilian sectors at the same time in one region of the Russian Federation. These isolates were analyzed with molecular epidemiologic techniques (spoligotyping, variable number tandem repeat [VNTR], rapid epidemiological typing [RAPET], IS6110) to characterize the role of Beijing isolates and molecular genotypic methods to determine rifampin, isoniazid, and multidrug resistance.

## Methods

### Population

All cultures of M. tuberculosis growing from February 1 to March 31, 2001, and May 14 to August 30, 2001, from five principal civilian and prison laboratories were analyzed: the Oblast Regional TB Laboratory (oblast laboratory), the Samara City Dispensary Laboratory no 1 (city dispensary), Samara City Hospital no. 1 (city hospital), Novokuibyshevsk Town Laboratory (Novokuibyshevsk laboratory), and Prison Colony no. 19 (prison colony). A single culture from each patient was analyzed. These five laboratories cover 40% of the population of this region with TB and are representative of the TB caseload in Samara Oblast. The oblast laboratory acts as the oblast reference/referral center; it has direct responsibility for rural areas, data collection for the oblast, and methodologic and organizational issues. The city dispensary receives cultures for drug-susceptibility testing from all five laboratories covering the outpatient caseload from the capital, Samara City; city hospital is the main inpatient facility. Novokuibyshevsk laboratory is the central TB laboratory; it serves a medium-sized industrial town. All patients with TB in the prison sector receive their inpatient treatment within the prison colony; the prison laboratory analyzes all positive cultures from prisoners. Gender, date of birth or age, date of culture, phenotypic drug-susceptibility results, prison status, and history of previous treatment were determined by direct questioning of each person and examining the medical and laboratory notes. The study was approved and supervised by the local drug-susceptibility testing and ethics steering committee.

### Drug Resistance

All sputum specimens were cultured on Lowenstein-Jensen media; specimens were coded so that those performing the laboratory analysis were unaware of the epidemiologic data. Rifampin, isoniazid, and multidrug resistance were determined genotypically. DNA was chloroform-extracted (20), and rifampin resistance was determined by using a nested PCR-amplification approach. The PCR product was reverse hybridized to probes immobilized on a membrane to detect mutations within the rpoB gene (21-23) (INNO-LiPA Rif.Tb; Innogenetics, Ghent, Belgium).

With the use of biotinylated primers, an inhouse macroarray was developed and used to identify mutations associated with isoniazid resistance in the genes inhA, katG, and ahpC (24). Briefly, crude bacterial lysates were prepared from each isolate as described above (20). With the use of published M. tuberculosis sequences, digoxigenin-labeled PCR products were generated in a multiplex PCR amplifying four genomic sequences associated with resistance to isoniazid: 251- and 232-bp regions of katG, including codon 315 and codon 463, a 241-bp region of aphC-oxyR; and a 265-bp region of the inhA locus, including the regulatory region. The sequences used to design the PCR primers were MTU06270 for katG, MTU16243 for aphC, and MTU66801 for inhA. Primer sequences were: tomkp 1 = GGCCCCGAACCCGAGGCTGC; tomkp2 = AACGGGTCCGGGATGGTGCCG; tomkp3 = GCCGACGAGTTCGCCAAGGCC; tomkp4 = ACGACGCCGCCGCCCATGCG; tomap1 = CCGCCGATGAGAGCGGTGAGC; tomap2 = CCACTGCTTTGCCGCCACCGC; tomip1 = CACCCGCAGCCAGGGCCTCG; and tomip2 = CGATCCCCCGGTTTCCTCCGG. PCR was conducted in a 25-µL reaction containing 2.5 µL 10X reaction buffer (Qiagen, Crawley,UK), 0.5 µL 2 mM dNTP (deoxynucleoside triphosphate; Pharmacia, Little Chalfont, UK), 20 µM each of the eight primers, 0.05 nmol dUTP (digoxigenin-labeled deoxyuridine triphosphate; Roche, Lewes, UK), 0.5 U of HotStartaq (Qiagen), and 1 µL DNA extract. Cycling was conducted with a PE2400 thermal cycler (Applied Biosystems, Foster City, CA) programmed to hold 95°C for 15 min then 30 cycles of 15 sec at 95°C, 30 sec at 60°C, and 60 sec at 72°C.

Eleven probes for the macroarray were diluted in water to 20 µM and applied to printed cells on a numbered nylon membrane (Osmonics, Minnetonka, MN). The oligonucleotides were UV-crosslinked for 1 min in an Amplirad UV box (GRI, Braintree, UK). Membranes were washed twice for 5 min in 0.5XSSC (1X SSC is 0.15 M NaCl plus 0.015 M sodium citrate) and air-dried. The individual arrays were then separated and placed in a 2.5-mL minifuge tube. These were stored in darkness at room temperature until use. A reverse hybridization procedure was used to interrogate the macroarray, and hybridization was visualized by detecting the digoxigenin label colorimetrically according to the manufacturer's instructions (Roche, Lewes, UK). Extensive precautions (including the use of a three-room PCR suite, dedicated equipment, and multiple positive and negative controls) were taken to avoid cross-contamination.

### Molecular Epidemiology

All cultures were analyzed by spoligotyping (15) and VNTR analysis (25) by using standard methods. For VNTR analysis, PCR-amplification was performed to identify the number of exact tandem repeats at five loci, A to E. PCR products were electrophoresed on a sieving agarose gel to determine the size of the product and compared against a standard to determine the number of exact tandem repeats at each locus. The result can be read as a numerical code that can be compared across large groups of isolates. RAPET was performed as described by Yates et al. (2). Fifty-five viable cultures were available for RFLP typing, targeting the IS6110 by using the internationally standardized protocol described by van Embden et al. (11).

## Results

### Drug Resistance

One hundred forty cultures were collected from the five sites, and genotypic analysis was performed in London by British and Russian scientists to determine rifampin and isoniazid resistance (Table 1). One hundred thirty-four cultures were successfully amplified (four cultures gave no amplification, and two cultures were identified as M. gordonae). Overall, 78 (58.2%) had mutations consistent with rifampin resistance. The Innolipa assay may underestimate true rifampin resistance by 5% to 7% (21,23). Detection of mutations, however, is indicative of true rifampin resistance in nearly all cases. Table 1 indicates the overall rate of rifampin resistance, the rate for each laboratory center, and the rate of molecular rifampin resistance as a fraction of each laboratory culture population.

**Table 1:** Molecular resistance to rifampin and isoniazid in *Mycobacterium tuberculosis* isolates with proportional analysis by center/laboratory, Samara Oblast, Russian Federation

Laboratory location	No. of isolates examined for rifampin resistance (n=130)^a^	No. of rifampin-resistant isolates (%)	No. of isoniazid-resistant isolates (%)^b^
			
All	134	78 (58.2)	66 (–)
Prison TB colony	45	36 (80.0)	27 (60.0)
City dispensary 1	11	4 (36.4)	4 (36.4)
City hospital 1	34	18 (52.9)	14 (41.2)
Novokubshev town laboratory	21	9 (42.9)	11 (52.4)
Oblast regional TB laboratory	19	11 (57.9)	10 (52.6)

^a^Of 140 cultures, 134 results were produced, but only 130 were available for results by center; 2 were *M. gordonae*, amplification failed for 4 cultures, and center could not be definitively identified for 4.  ^b^Of 140 cultures, amplification products that could be evaluated were obtained from 128 cultures.

A macroarray was used to identify isoniazid resistance in 128 cultures that could be evaluated (amplification products were absent in 6 cultures successfully amplified for rpoB). When this method was used, 66 (51.6%) isolates were isoniazid resistant, and 62 were sensitive. This technique only detects approximately 75% to 90% true resistance so the rate of resistance is underestimated. Table 1 indicates the proportions of isoniazid-resistant cultures from each center; most came from the prison TB laboratory. Patient age and being a prisoner were significant risk factors for rifampin but not isoniazid resistance; previous treatment was a risk factor for resistance to both drugs (Table 2).

**Table 2:** Patient factors associated with rifampin- and isoniazid-resistant isolates^a^

Factor	Rifampin resistance (n=78)	Isoniazid resistance (n=66)
Age	p<0.05^b^	p>0.05
Known prisoner	RR 1.68,^b^ CI 1.29 to 2.18	RR 1.36, CI 0.97 to 1.89
Known previous treatment	RR 1.71,^b^ CI 1.07 to 2.74	RR=2.04,^b^ CI 1.01 to 4.10

^a^RR, risk ratio; CI, confidence interval. ^b^Statistically significant.

### Beijing Family and Molecular Epidemiology

Of the 140 original samples, 138 produced a spoligotype profile. The samples were divided into 27 groups with 16 individual isolates and 10 clustered groups: 5 clusters containing 2 isolates, 2 with 3 isolates, 1 with 4 isolates, and 1 with 8 isolates. The 10th cluster accounted for 94 (68.1%) of the 138 cultures and comprised the characteristic Beijing strain (i.e., binding occurred only to the final nine spoligotype probes). When VNTR typing was used, 136 isolates (4 did not amplify) were divided into 25 groups: 13 were individual profiles with 4 clusters containing 2 isolates, 2 clusters of 3 isolates, 1 cluster of 4 isolates, 2 clusters of 5 isolates, 1 cluster of 9 isolates, 1 cluster of 13 isolates, and a dominant cluster of 73 strains. Combining spoligotyping and VNTR typing divided the 138 strains into 44 types in total.

The proportion of Beijing isolates compared to the total number of cultures submitted by each site was calculated (Table 3). Both the total number and highest proportion of Beijing family cultures came from the prison and the oblast TB dispensary, which would be inclined to have complicated cases, including ones in former prisoners.

**Table 3:** Proportion of Beijing family in isolates of *Mycobacterium tuberculosis* with distribution by center/laboratory, Samara Oblast, Russian Federation

Location	No. of isolates available for typing (n= 138)^a^	No. of Beijing family isolates (%)
		
All	138	94^b^ (68.1)
Prison TB colony	49	40 (81.6)
City dispensary 1	11	6 (54.5)
City hospital 1	34	18 (52.9)
Novokuibyshevsk town laboratory	21	12 (57.1)
Oblast regional TB laboratory	19	14 (73.7)

^a^Of 140 cultures, 138 were available for spoligotyping (2 were not multiresistant TB); however, 4 cultures could not be attributed to a center. ^b^38 cultures were evaluated, but 4 could not be attributed to a specific center, i.e., 134 attributable cultures

### Beijing Strains and Drug Resistance

Overall, 65 (69.1%) of 94 Beijing family cultures were rifampin resistant (Table 4); the highest proportion of rifampin-resistant Beijing isolates (31/38 or 81.6%) occurred in the prison. Proportions at the other centers varied from 60.0% to 77.8%.

**Table 4:** Number and proportion of rifampin resistant isolates within Beijing family at each center

Location	No. of Beijing isolates	No. of Beijing isolates resistant to rifampin (%)	No. of isolates resistant to rifampin	% of rifampin-resistant isolates
All	94	65 (69.1)	78	83.3
Prison TB colony	40	31 (77.5)	36^a^	86.1
City hospital 1	18	14 (77.8)	18	77.8
Novokuibyshevsk town laboratory	12	7 (58.3)	9	77.8
Oblast regional TB laboratory	14	10 (71.4)	11	90.9

^a^Four gave no amplification; numbers too small to evaluate for city dispensary l.

High rates of rifampin resistance were seen in Beijing isolates from all centers. Overall, the proportion of Beijing isolates with rifampin resistance was over twice that of non-Beijing isolates (69.1% vs. 29.5%). The Beijing cluster of 94 isolates was subtyped by using VNTR (Table 5); 92 isolates were successfully analyzed, and no amplification occurred in 2 isolates. Overall, the 91 Beijing isolates were subdivided into 13 groups with 8 individual VNTR types and 2 different clusters of 2 isolates, 1 cluster each of 3 isolates and 1 of 9 isolates. The 13th cluster contained 68 isolates (VNTR 42435) or 72.3% of the total number of Beijing isolates seen. Most isolates within this VNTR type were rifampin resistant: 50 (73.5%) of 68 were resistant, 16 (23.5%) were sensitive, and 2 had equivocal results. Within this dominant VNTR type, the proportion of the type that was rifampin resistant by institution was calculated (Table 6). Within the prison and oblast dispensary 90% to 91% of the strains of the VNTR 42435 type were rifampin resistant.

**Table 5:** Variable number tandem repeat (VNTR) analysis of Beijing isolates (n=91) and comparison with rifampin sensitivity

VNTR	Total in each VNTR type	No. of rifampin resistant in each type	No. of rifampin sensitive
12435	3	3	0
12534	9	8	1
12535	1	1	0
22232	2	0	2
22435	1	0	1
32413	1	-	–^a^
32433	1	0	1
32435	1	0	1
42234	1	0	1
42434	2	0	2
42435	68	50	16 (2 mixed=nonreadable)^a^
42436	1	1	0
42532	1	1	0

^a^ In three, no amplification of *rpoB* gene or mixed reaction.

**Table 6:** Proportion of dominant variable number of tandem repeat (VNTR) isolates of type 42435 known to be resistant or sensitive, by institution

Location	Proportion (n=68)	No. of VNTR type resistant to rifampin (%)^a^	Proportion of VNTR type sensitive to rifampin (%)
			
Prison TB colony	30	27 (90)	3 (10)
CD1	5	3 (60)	2 (40)
City hospital 1	7	3 (42.9)	4 (57.1)
Novokuibyshevsk town laboratory	12	7 (58.3)	5 (41.7)
Oblast regional TB laboratory	11	10 (90.9)	1 (9.1)

^a^No *rpoB* amplification in one culture in this group.

Of the 55 viable isolates that were RFLP-IS6110 fingerprinted, 23 were members of the main VNTR 42435 group (Figure). A small number of similar but distinct isolates were seen across the sites, including the W148 strain seen in Siberian prisons and elsewhere in the former Soviet Union (18). This observation was confirmed by RAPET typing (data not shown).

**Figure:**
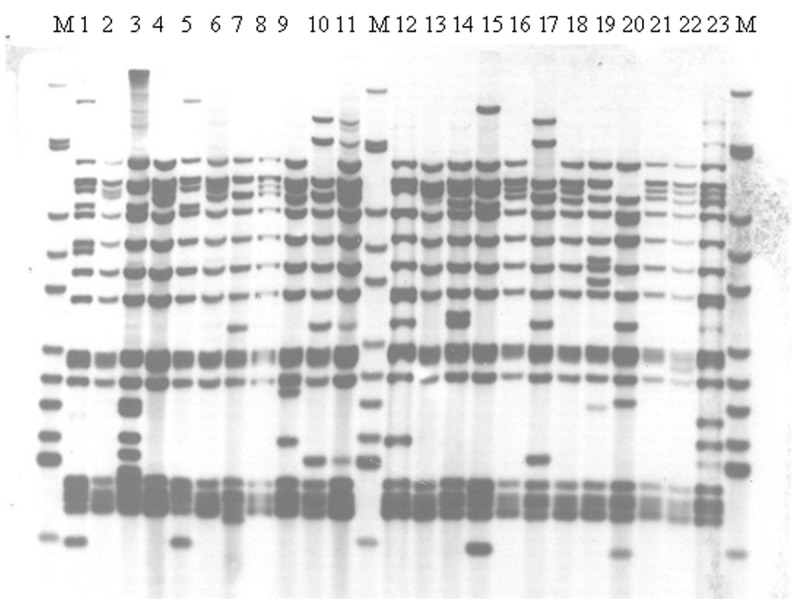
IS6110 restriction fragment length polymorphism analysis of isolates from a dominant variable number tandem repeat group of Beijing family isolates at all sites, Samara, Russia. M indicates Mycobacterium tuberculosis strain MT14323. Isolates were from all five sites including the prison (tracks 13, 16,18, and 20).

## Discussion

This analysis is part of an ongoing program to develop the capacity to accurately determine drug resistance and monitor the epidemiology of drug-resistant TB in both civilian and prison sectors in Russia. For multidrug-resistant TB, cure rates are lowered and infectious patients remain sputum smear-positive longer, increasing the probability that others will be infected.

As part of this program, we initiated a collaborative study in Samara, Russia, 1 of 89 regions or oblasts within the Russian Federation. In 2000, the new case rate in Samara was 87.6/100,000 (2,890 cases) in a population of 3,308,000 (including nonresidents and prisoners) with a death rate of 13.6 /100,000 (443 cases) (9,10). TB appears to be a problem in all Russian prisons including those in Samara, where the total number of TB patients at the time of the study was 1,800 cases (~34% of all registered TB patients in the region) (9,10). Because of an increasing number of intravenous drug users in the region, the problem of coinfection with HIV is likely to become increasingly important as the number of HIV-positive persons increases (>12,000 HIV-infected persons were registered in Samara Region in May 2001).

When molecular genotypic resistance analysis was used, 58.2% of cultures were rifampin resistant, and at least 51.6% were shown to be isoniazid resistant. Within this population, there were 123 molecular results for both rifampin and isoniazid, and 55 cases were definitely multidrug-resistant TB (55/123 or 44.7%); and 24 were possible cases (i.e., resistant to rifampin but sensitive to isoniazid with genotypic methods). These rates are high for both rifampin resistance and multidrug-resistant TB. Nevertheless, care must be taken in interpreting these results: they are likely to be overestimates since cultures were derived from new and chronic cases. Very high rates of rifampin resistance were seen in cultures drawn from the prison TB colony (80%), and 35 (79.5%) of 44 patients with known rifampin-resistant cases had been in prison before. Being a prisoner and patient age were risk factors for rifampin resistance (but not for isoniazid resistance). Previous treatment was a risk factor for both rifampin and isoniazid resistance.

It remains unclear whether rifampin resistance is being introduced into the oblast prison from the pretrial centers or developing within prison because of interrupted therapy caused by poor adherence to treatment or release before treatment is completed. Previous treatment has been shown to be a risk factor for drug resistance (1,2).

Molecular epidemiologic techniques can help monitor the spread of TB isolates. These techniques will be of particular value where drug resistance is common and where strains such as the Beijing family appear more commonly associated with the type of drug resistance likely to lead to therapeutic failure and prolonged infectivity. In this study, rapid PCR-based techniques such as spoligotyping were used; although less discriminating than IS6110-RFLP analysis, PCR techniques permitted safe, rapid analysis of a specific family of strains. Spoligotyping's ability to discriminate was greater than that of VNTR; in combination, the techniques divided the isolates into 43 groups, indicating the benefits of combining these systems. Spoligotyping demonstrated a high proportion (94 [68.1%] of the 138 cultures) of the characteristic Beijing family of strains, which has been previously associated with drug resistance.

Some researchers have argued that the Beijing genotypes may have a selective advantage over other genotypes; BCG-induced immunologic protection may not protect against this strain, which would be an "escape mutant" (17). In that study in Vietnam, although the Beijing isolates were occurring more frequently among those vaccinated with BCG, this difference was not statistically significant (17). In that study, more Beijing isolates occurred in younger age groups, suggesting possible recent transmission; in our analysis, patients with Beijing isolates were younger than those with non-Beijing isolates (data not shown), which might support this conjecture. Other researchers have argued against any selective advantage, pointing out that Beijing isolates have spread widely in the United States, where BCG vaccination is not used (18). Nevertheless, BCG vaccination may accelerate the dominance of this family in regions once the strains have been introduced. The Russian Federation TB Service has a comprehensive program of BCG vaccination, which might create a selection pressure. Analysis using VNTR also demonstrated a dominant VNTR type containing 68 (72.3%) isolates (VNTR 42435) of the total number of Beijing isolates. A small number of similar but distinct isolates were seen on RFLP-IS6110 fingerprinting, including the W148 strain (17 band) seen in Siberian prisoners and elsewhere in the former Soviet Union (18). In 1998-2000, this strain was seen in 190 prisoners with multidrug-resistant TB in a prison in Tomsk, Western Siberia (18,19). None of the Samaran prisoners were from Siberia.

Another probable reason for the successful expansion of the Beijing family is its association with multidrug-resistant TB; Beijing types such as W4 and "210" appear widespread in China but are fully susceptible to drugs (18). Low cure rates of smear-positive prisoners would lead to prolonged infectivity. The oblast prison, in particular, has a high proportion of Beijing isolates and a high rate of rifampin resistance, suggesting that the Beijing family is dominant there and accounts for a large proportion of resistance. Nevertheless, drug resistance per se cannot be the only explanation for the success with these isolates. The widespread population movements during the first half of the 20th century may have helped to distribute these isolates into new communities, which were then subsequently selected for by the introduction of a comprehensive BCG vaccination policy and by the later development of drug resistance. Concluding that Beijing family isolates have undiscovered advantages is also reasonable. In this study a large proportion of the Beijing isolates were of the same VNTR type (42435), but little is known of any differences in biologic function that might be related to different types. A recent study has demonstrated that the coding sequence Rv3710 (leuA) encodes the production of active alpha-isopropylmalate synthase. Within the sequence lies the locus of VNTR 4155, and this may have a modifying role on the function of the enzyme (26).

High-quality national or regional drug resistance surveys are needed in other parts of the Russian Federation. Such surveys would lead to a clearer understanding of the true level of drug resistance and in turn facilitate clinical management and permit better empirical treatment strategies. Genotypic techniques are of value in determining rifampin resistance and likely multidrug-resistant TB. Although expensive compared to drug-susceptibility testing on solid media, genotyping techniques may be justified in populations with high levels of multidrug-resistant TB and high rates of concurrent HIV. Further analysis is required to confirm the spread of the Beijing family and determine whether it is imported into the prison and spread within the prison community.
